# 

**Published:** 1881-06-20

**Authors:** 


### EDITORIALS AND MISCELLANEOUS.

*Receipts of Subscribers* postponed until next issue.

DR. H. L. BARTLETT was appointed by the American Medical Association as a delegate to the British Medical Association.

*Canned Beef not Safe.*—Eight cases of poisoning from eating canned beef has been recently reported in New York city.

*U. S. Dispensatory.*—There is no new U. S. Dispensatory, as one of our correspondents seems to suppose. The National Dispensatory is now being used in its stead.

*Richardson & Co., Wholesale Druggists, St. Louis.*—We invite special attention to the advertisement of this excellent and reliable house. Their combination called CELERINA is attracting attention. Read the advertisement.

*Lambert & Co., Manufacturing Chemists, St. Louis.*—This reliable and enterprising house commences an advertisement of their antiseptic preparation LISTERINE, in this issue of our Journal. It is an excellent preparation. See the advertisement.

*Dr. Sayer Sued for Malpractice.*—It is stated that Dr. Sayer, of New York, made the following prescription for a lady, who, having a train of hysterical symptoms of a peculiar character, attributed them to the medicine—

℞ Aloes........................................................ ʒjss.

Ext. hyoscyamus,

Ext. nux vomica,.......................................... aa ʒss.

M. Ft. Pills No. thirty. One to be taken every four hours.

The patient took four of the pills in less time than that prescribed. The testimony, however, showed that the symptoms were due to hysteria, and not to the medicine.

*Birth Rate Declining.*—It is affirmed that there is a decline in the birth-rate in this country, a fact not heretofore noticed in our census returns. While there is a rapid increase of population in the United States, it is due in a greater proportion than formerly to immigration; the ratio of increase from births being less than that indicated by the census in the earlier history of the country. There are two reasons assigned for the decline of the birth-rate—the one, an increase of menstrual disorders consequent upon the educational methods and fast living of the day, by which brain culture and precocious habits are unduly developed at the expense of physical health and strength; the other, the very perceptible fact that families are numerically smaller than in years past, indicating the use of various methods and means to prevent conception. This is the evil, which, of late years, has alarmed the statesmen of France, in which country it has been, perhaps, more prevalent than elsewhere, and has given rise to serious apprehensions for the future growth and prosperity of the Empire.

Dr. Goodell, in an address before the Medical and Chirurgical Society of Maryland, attributes this growing evil to "*The faulty system of female education, the decay of home-life, etc., and the unwillingness of our women to become mothers.*"

